# Real-World Eligibility for FLAURA, FLAURA2, and MARIPOSA in Patients with EGFR-Mutated Advanced NSCLC: A Retrospective Single-Center Study

**DOI:** 10.3390/cancers18213480

**Published:** 2026-10-28

**Authors:** Silvia Marchesi, Paolo Ambrosini, Chiara Cavalli, Rebecca Romanò, Alessia Vendittelli, Stefano Natangelo, Giuseppe Nardo, Margherita Piccinelli, Daniele Lorenzini, Marta Brambilla, Laura Mazzeo, Teresa Beninato, Giulia Corrao, Federica Perrone, Rosa Maria Di Mauro, Andra Diana Dumitrascu, Claudia Proto, Arsela Prelaj, Monica Ganzinelli, Filippo Guglielmo Maria De Braud, Giuseppe Lo Russo, Mario Occhipinti

**Affiliations:** 1 Department of Medical Oncology, Fondazione IRCCS Istituto Nazionale dei Tumori di Milano, Milan 20133, Italy; 2 Department of Advanced Diagnostics, Fondazione IRCCS Istituto Nazionale dei Tumori, Milan 20133, Italy

**Keywords:** *EGFR*-mutated NSCLC, randomized controlled trials, real-world practice

## Abstract

Introduction: First-line treatment options for *EGFR*-mutated NSCLC have expanded beyond osimertinib monotherapy following the FLAURA2 and MARIPOSA trials, which demonstrated improved long-term outcomes with combination strategies, albeit with increased toxicities. Given that randomized controlled trials (RCTs) typically enroll highly selected patients (pts), evaluating the representativeness of these cohorts in real-world (RW) practice is essential to inform RCT applicability. Methods: We retrospectively identified patients with EGFR-mutated advanced NSCLC treated with first-line osimertinib at Fondazione IRCCS Istituto Nazionale dei Tumori, Milan, between January 2019 and August 2025. Clinical characteristics were collected, and eligibility for FLAURA, FLAURA2, and MARIPOSA was assessed according to the respective trial criteria. For survival analyses, patients were classified as trial-eligible or trial-ineligible according to FLAURA eligibility criteria, as all patients received first-line osimertinib. Results: Overall, 135 pts were included in the analysis; median age was 67 years (IQR 57–76), 17% had ECOG PS 2, and 11% harbored uncommon EGFR mutations. Only 31 patients (23%) met eligibility criteria for all three trials, whereas 91 (67%) were ineligible for all. The most frequent exclusion criteria included symptomatic CNS disease or spinal cord compression, ECOG PS 2, and EGFR mutation assessment by cytology or liquid biopsy. Both real-world progression free survival (rwPFS) and overall survival (OS) were numerically longer in trial-eligible than trial-ineligible patients (20.1 vs. 18.2 months and 29.9 vs. 22.3 months, respectively). Patients with negative prognostic characteristics such as ECOG PS ≥ 2, uncommon *EGFR* mutations, and liver metastases had significantly shorter OS, while other predefined risk factors showed only numerical trends. Conclusions: A substantial proportion of patients treated with first-line osimertinib in routine practice would not have met the eligibility criteria of pivotal EGFR-mutated NSCLC trials. Trial-ineligible patients had numerically shorter survival, likely reflecting adverse baseline characteristics. These findings highlight the limitations of direct extrapolation of RCT results to real-world populations.

## Simple Summary

This study investigated whether patients with advanced lung cancer with mutations in the epidermal growth factor receptor (EGFR) gene who received osimertinib in routine clinical practice would have been eligible to participate in three major clinical trials that evaluated first-line treatments in this setting. We reviewed the clinical records of 135 patients and assessed their characteristics against the eligibility criteria of the three trials. Only 31 patients (23%) met the criteria for all three trials, while 91 (67%) would not have qualified for any of them. The most common reasons for exclusion included symptomatic brain involvement or spinal cord compression and poor general health. Patients who met the eligibility criteria of the FLAURA trial also showed numerically longer survival than those who did not, although this difference should be interpreted cautiously. These findings highlight that patients treated in everyday clinical practice often differ from those enrolled in clinical trials. Understanding these differences can help clinicians interpret trial results more carefully and make treatment decisions that better reflect the characteristics and needs of individual patients.

## 1. Introduction

Epidermal growth factor receptor (*EGFR*) mutations occur in approximately 20% of patients with metastatic non-small cell lung cancer (NSCLC) [1]. First-line osimertinib has become the standard of care for patients with advanced or metastatic NSCLC harboring activating common *EGFR* mutations such as exon 19 deletion or L858R mutation, following the results of the pivotal phase III FLAURA trial, which demonstrated a significant progression-free survival (PFS) and overall survival (OS) benefit over gefitinib or erlotinib [2]. Recently, the therapeutic landscape for first-line *EGFR*-mutated NSCLC has expanded, as novel combination strategies were introduced in clinical practice based on the results of two pivotal phase III trials. Among these, the combination of osimertinib and platinum-based chemotherapy demonstrated PFS and OS superiority compared to osimertinib alone in the FLAURA2 trial [3,4], as well as the novel dual inhibition approach with amivantamab and lazertinib in the MARIPOSA trial [5,6]. Combination strategies are nevertheless burdened by worse toxicity profiles due to the concomitant use of multiple therapeutic agents. When choosing between the three currently available regimens, clinicians must consider several factors, including patients’ general conditions, comorbidities, disease extent, and the presence of negative prognostic factors.

Randomized controlled trials (RCTs) often include highly selected populations that may not reflect the heterogeneity of real-world (RW) clinical practice. This discrepancy raises concerns about the external validity and generalizability of trial results [7]. Several retrospective studies have previously assessed the proportion of RW patients who would have been eligible for the FLAURA trial, demonstrating that only a minority of those treated with first-line osimertinib would have met the inclusion criteria of FLAURA [8]. Furthermore, notable differences in OS were reported between eligible and ineligible patients [9,10].

The present retrospective study analyzes a RW *EGFR*-mutated NSCLC population to assess differences in baseline characteristics compared to trial populations, as well as FLAURA, FLAURA2, and MARIPOSA trial eligibility of the RW population. As combination regimens are poised to enter clinical practice, it becomes crucial to evaluate how representative the trial populations are of the patients seen in RW settings.

## 2. Materials and Methods

### 2.1. Study Population

Patients with *EGFR*-mutated advanced or metastatic NSCLC who received first line osimertinib at Fondazione IRCCS Istituto Nazionale dei Tumori (INT) of Milan from January 2019 to August 2025 were included in this study. Patients who received prior 1st or 2nd generation *EGFR* TKI but switched to osimertinib due to toxicity or patients who initiated osimertinib administration in another institution and were subsequently referred to INT were excluded. The data cut-off date was set on 15 November 2025. All patients were enrolled in the APOLLO 11 study, an Italian multicenter, observational, retrospective/prospective study involving patients with a diagnosis of advanced lung cancer [11].

### 2.2. Study Design

This is a retrospective single-center study aiming to analyze the distribution and characteristics of an RW population of patients with advanced/metastatic *EGFR*-mutated NSCLC treated with osimertinib, and to compare them to FLAURA, FLAURA2, and MARIPOSA trial populations and eligibility criteria. Patients’ information including demographic, clinical, and histopathological characteristics were retrospectively extracted from electronic medical records and APOLLO 11 Case Report Forms (CRFs). Treatment response and duration and subsequent therapeutic treatments were collected as well. Furthermore, Charlson Comorbidity Index was calculated for each patient to estimate the burden of comorbidities. The inclusion and exclusion criteria of the FLAURA, FLAURA2, and MARIPOSA trials were retrieved from the publicly available trial protocols included in the Supplementary Materials of the respective publications, together with the final trial reports [2,3,5]. Each criterion was assessed retrospectively based on the information available in the medical record at the time of osimertinib initiation.

### 2.3. EGFR Testing

*EGFR* mutation detection was performed according to institutional standard procedures and international guideline recommendations. *EGFR* alterations were assessed on histological or cytological tumor samples or by circulating tumor DNA (ctDNA) analysis, when tissue was unavailable. Different detection methods were used, including single gene PCR, and next generation sequencing (NGS) with Hot-spot Cancer Panel and Oncomine Comprehensive Assay (OCA) Plus, depending on tumor cellularity, sample availability, and panel accessibility. Co-mutations were reported when available for samples analyzed with NGS. Tumor mutational burden (TMB) determination was available for a subset of samples analyzed with OCAplus panel.

### 2.4. Outcomes

The primary endpoint of this study was to assess the proportion of RW patients eligible for the FLAURA, FLAURA2, and MARIPOSA trials. Secondary endpoints included the reasons for trial exclusion, defined as the percentage of patients not meeting each inclusion/exclusion criterion; comparison of baseline characteristics of RW population and RCT cohorts; median OS (mOS) in the entire population and by trial eligibility; median real-world PFS (rwPFS) in the entire population and by trial eligibility; disease control rate (DCR); and objective response rate (ORR). As all patients received first-line osimertinib, comparisons of rwPFS and OS according to trial eligibility were based exclusively on the FLAURA eligibility criteria. OS was calculated from time of osimertinib start to death from any cause, with patients alive at last follow-up censored. rwPFS was defined as the time from osimertinib initiation to the first documented real-world progression according to RECIST criteria or death from any cause, whichever occurred first. Patients without a documented event were censored at the date of the last available imaging assessment. Radiological assessments were performed according to routine clinical practice, without prespecified assessment intervals. The radiological response was RECIST-evaluated retrospectively, based on documentation in the available records and radiology reports. ORR was defined as the proportion of patients achieving complete or partial response, while DCR included complete response, partial response, and stable disease. Oligoprogression was defined as an increase in the size of one or a limited number of previously identified lesions that did not meet RECIST 1.1 criteria for progressive disease and was managed with local treatment while continuing osimertinib. In these patients, oligoprogression was not considered an rwPFS event; the event date was defined as the date of subsequent RECIST-defined progression or death.

### 2.5. Statistical Methods

OS and rwPFS were determined using the Kaplan–Meier method. Survival curves were compared using the log-rank test. Subgroup analyses were performed according to clinically relevant variables, including Eastern Cooperative Oncology Group Performance Status (ECOG PS) (0–1 vs. ≥2), age (<65 vs. ≥65 years), sex, smoking status (current vs. former vs. never), presence of brain metastases, presence of liver metastases, *EGFR* mutation subtype (exon 19 deletion vs. L858R), TP53 mutation status, and PD-L1 expression levels. All subgroup analyses were exploratory and unadjusted. All statistical tests were two-sided, and a *p*-value < 0.05 was considered statistically significant. Statistical analyses were performed using RStudio v. 2024.09.1+394.

## 3. Results

### 3.1. Patients’ Cohort

**Figure 1:**
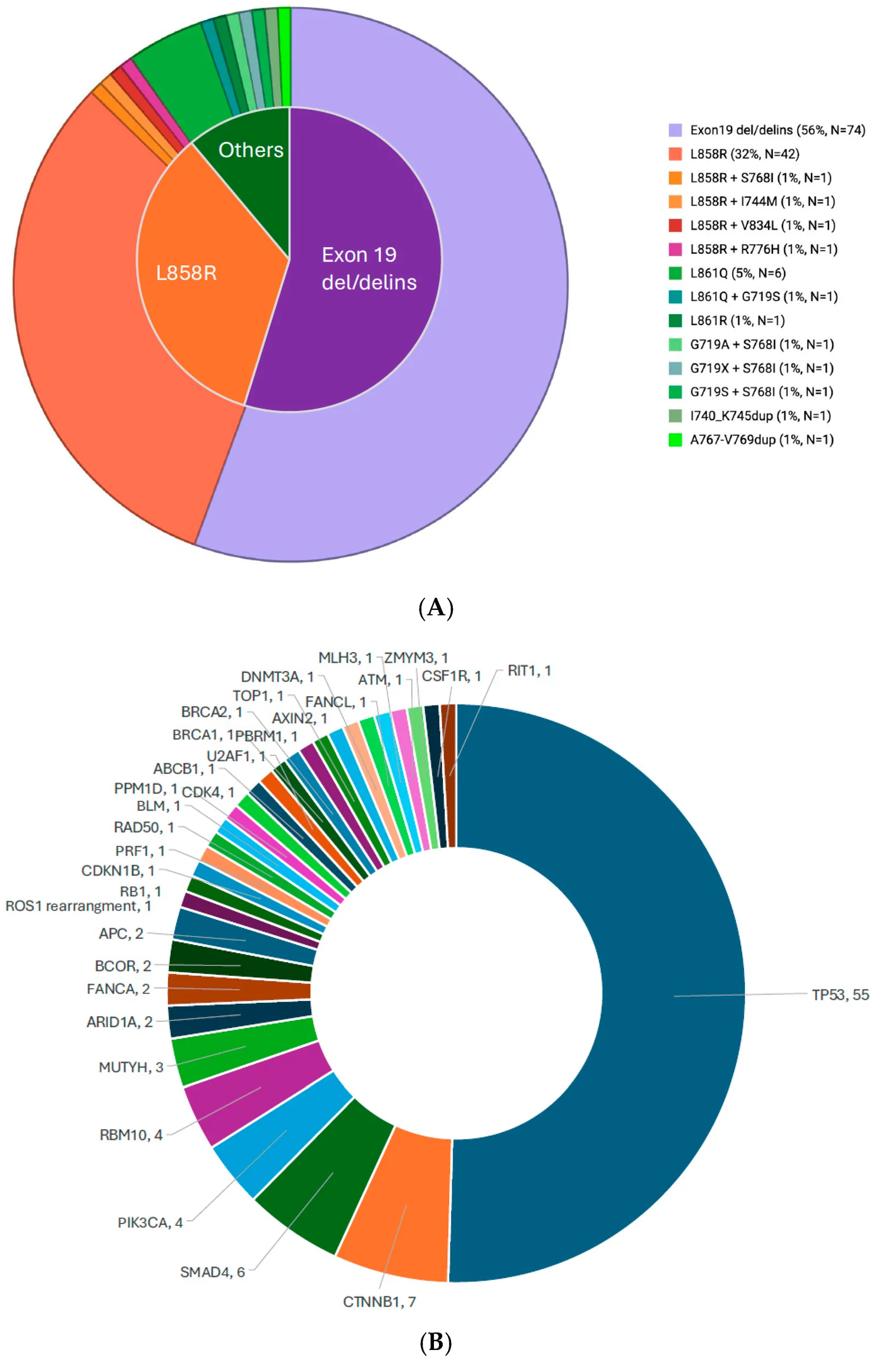
(**A**): *EGFR* mutation type and frequency. (**B**): Concomitant mutations and frequency. Concomitant mutations are available in 93 pts; different panels were used. Among these, no co-mutation *n* = 19; 1 co-mutation *n* = 50, 2 co-mutations *n* = 14, 3 co-mutations *n* = 8, 4 co-mutations *n* = 2. One patient had ROS1 rearrangement identified by fluorescence in situ hybridization (FISH).

A total of 135 patients met the inclusion criteria and were included in the analysis. Two-thirds of the patients were female (*n* = 90, 67%); among these, 23 patients (17%) had been previously diagnosed with early-stage disease and had received a curative-intent treatment (Supplementary Table S1). Smoking status was available for 130 patients: 65 (48%) were never-smokers and 65 (48%) were current or former smokers. Most patients had adenocarcinoma (*n* = 127, 94%). *EGFR* exon 19 deletion was the most common molecular alteration (*n* = 74, 55%), followed by L858R mutation (*n* = 46, 34%). Molecular profile including co-mutations is represented in Figure 1. In 97 patients (72%), PD-L1 Tumor Proportion Score (TPS) was <50% (unavailable in 18 cases); TMB was available for 49 patients, with a median value of 5.86 mut/Mbp (range 0.95–15.14). At osimertinib start, stage IVB disease was the most frequent (*n* = 101, 75%), and 36% of the patients had at least three metastatic sites. Among the 48 pts with brain metastases, 16 were treated locally before osimertinib start (radiotherapy *n* = 12; surgery *n* = 2; surgery and radiotherapy *n* = 2). Median age at osimertinib start was 67 [IQR 57–76] and median Charlson Comorbidity Index was 8 (range 6–13), mainly dependent on age and presence of a metastatic cancer. ECOG PS at osimertinib start was 0 in 46 patients (34%), 1 in 65 (48%), ≥2 in 23 (17%). Patients’ characteristics are reported in Table 1.

### 3.2. Baseline Characteristics Comparison Between Trials Arms and RW Cohort

Compared to trial populations, patients in the RW cohort were older (median age 67 years vs. 61–64 across FLAURA, FLAURA2, and MARIPOSA). The distribution of disease stage was similar, with metastatic disease observed in 95% of RW patients, consistent with the 95–97% reported in RCTs. However, the RW cohort included a clinically relevant proportion of patients with poorer performance status, with 17% presenting ECOG PS ≥ 2, whereas such patients were virtually absent in trials (0–<1%). Differences were also observed in *EGFR* mutation patterns: uncommon mutations were present in 11% of RW patients (15/135), while these were not represented in the trial populations. Conversely, the proportion of exon 19 deletions was slightly lower in RW (55% vs. ~60–63%), as was L858R (34% vs. ~37–40%). The prevalence of brain metastases was comparable between RW and RCT cohorts (36% vs. 19–42%). Table 2 reports data available for the different arms of RCTs and RW cohort.

### 3.3. Trials Eligibility

**Figure 2:**
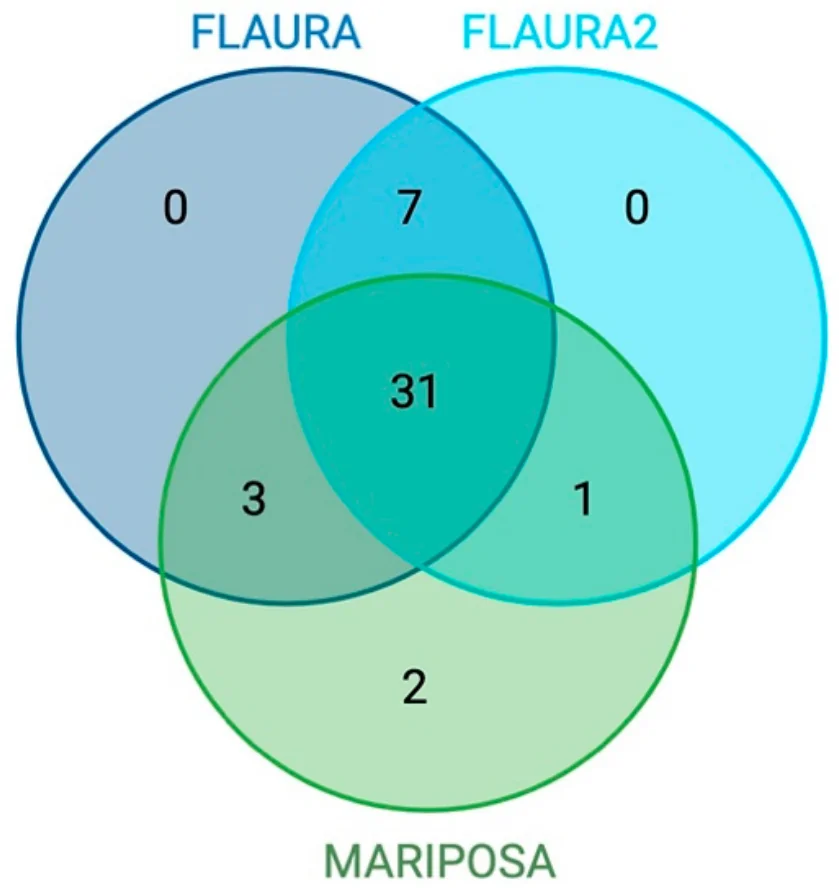
Overlap in patient eligibility across FLAURA, FLAURA2, and MARIPOSA trials. Venn diagram showing the number of patients meeting the eligibility criteria for each trial and their overlap. Of 135 patients assessed, 44 were eligible for at least one trial, including 31 patients eligible for all three trials. Seven patients were eligible for both FLAURA and FLAURA2 but not MARIPOSA, three for FLAURA and MARIPOSA but not FLAURA2, and one for FLAURA2 and MARIPOSA but not FLAURA. Two patients were eligible for MARIPOSA only, whereas no patient was eligible exclusively for FLAURA or FLAURA2. Ninety-one patients were not eligible for any of the three trials.

**Figure 3:**
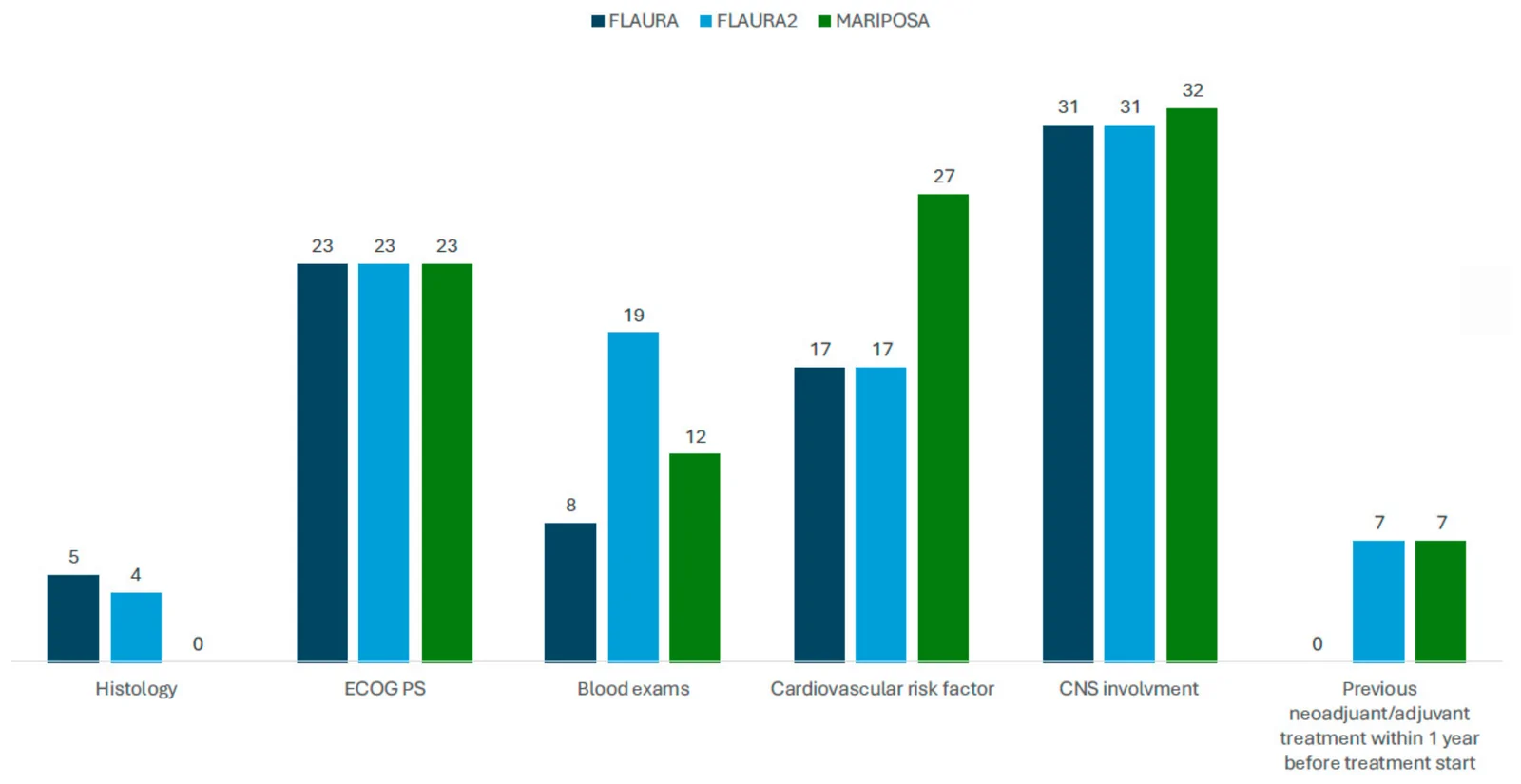
Comparison between main reasons for trial exclusion between FLAURA, FLAURA2, and MARIPOSA. Abbreviations: ECOG PS = Eastern Cooperative Oncology Group Performance Status; CNS = central nervous system.

When assessing RW cohort according to trials inclusion and exclusion criteria of the RCTs, only a minority of the patients (*n* = 31, 23%) could be enrolled in the three trials, while 91 patients (67%) were ineligible for all three trials (Figure 2). Among the ineligible patients for the FLAURA trial, 53 patients did not meet one criterion, 26 were excluded for two criteria, 13 for three criteria, and 2 for four criteria. Similar data are reported when applying the FLAURA2 criteria. In detail, 47 patients were excluded due to one criterion, 24 due to two, 19 due to three, and 6 due to four criteria. The most frequent reason for exclusion for both FLAURA and FLAURA2 trials was the presence of symptomatic CNS localization or spinal cord compression (*n* = 31), ECOG PS ≥ 2 (*n* = 23), and *EGFR* mutation identification on cytological sample/liquid biopsy (*n* = 19). When analyzing the MARIPOSA trial, the number of not-met criteria were one in 47 patients, two in 25 patients, three in 14 patients, four in 11 patients, and five in 1 patient. The most frequent reasons for exclusion from MARIPOSA trial were the presence of symptomatic or steroid-required brain metastasis (*n* = 30), concomitant cardiovascular disease (*n* = 27), and ECOG PS ≥ 2 (*n* = 23). The main reasons for trial exclusion are represented in Figure 3 and all detailed unmet criteria are reported in Supplementary Table S2A–C.

### 3.4. Osimertinib Outcomes

**Figure 4:**
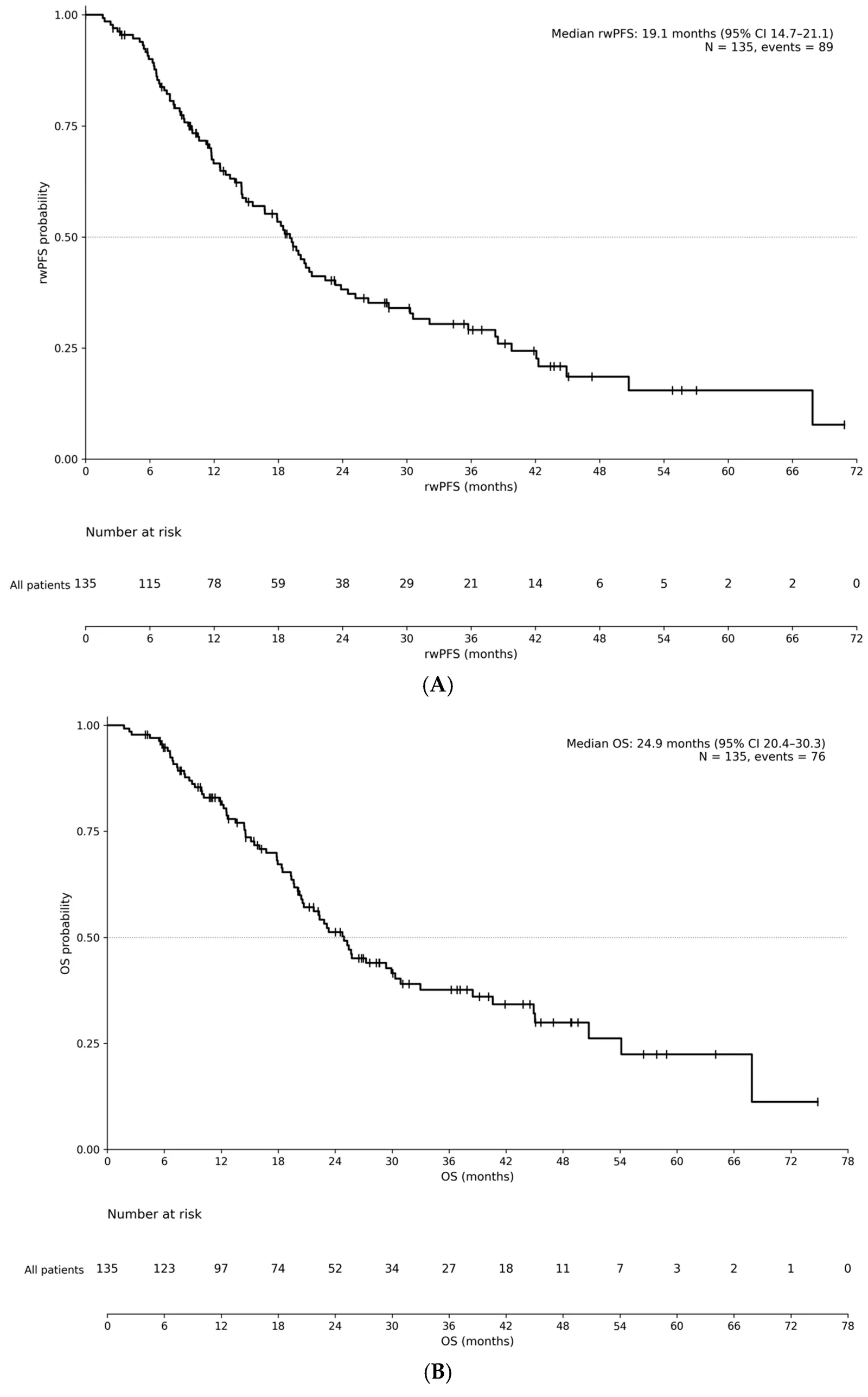
Kaplan–Meier curves representing (**A**) rwPFS and (**B**) OS of osimertinib RW cohort.

In the RW cohort, with a median follow-up of 38.5 months, rwPFS and mOS with first-line osimertinib were 19.1 (95% CI: 14.7–21.1) and 24.9 (95% CI: 20.3–30.3) months, respectively (Figure 4A,B). ORR was 70% and DCR was 98%. Only three patients experienced disease progression at the first imaging evaluation. During treatment, 34 patients (25%) experienced oligoprogression and received locoregional treatments, while continuing osimertinib. Thirty-one patients died during first-line treatment without radiological progression, while among the 69 patients who experienced unequivocal disease progression, 41 underwent further systemic treatment (3 lines *n* = 16; 4 lines *n* = 8). Patients with ECOG PS ≥ 2 had a numerically shorter rwPFS and significantly shorter OS compared with patients with ECOG PS 0–1 (rwPFS: 11.3 vs. 19.4 months, *p* = 0.052, OS 18.4 vs. 25.7 months, *p* = 0.010) (Supplementary Figure S1A,B). Longer rwPFS was also reported in patients without liver metastasis when compared to patients with liver metastasis (20.1 vs. 11.3 months, *p* = 0.006), as well as OS (25.4 vs. 19.4 m, *p* = 0.046) (Supplementary Figure S1C,D). Compared with male pts, female pts had a longer rwPFS (20.9 m vs. 13.1 m, *p* = 0.024) and OS (27.3 m vs. 20.4 m, *p* = 0.077) (Supplementary Figure S1E,F). Furthermore, pts with common *EGFR* mutation had better rwPFS (20.1 m vs. 11.3 m, *p* = 0.004) and OS (25.7 m vs. 17.9 m, *p* = 0.020) when compared to those with uncommon *EGFR* mutations (Supplementary Figure S1G,H).

When evaluated according to other predefined risk factors, such as age ≥ 65 years and presence of brain metastases, OS and rwPFS were lower in the corresponding subgroups (Supplementary Figure S2A–D). Conversely, patients with concurrent TP53 mutation had a numerically longer rwPFS and OS than those without (Supplementary Figure S2E,F). When stratified by PD-L1 expression, patients with PD-L1 ≥ 50% had numerically shorter rwPFS and OS compared to those with PD-L1 0–49% (rwPFS 12.0 m vs. 19.3 m, *p* = 0.306; OS: 21.7 vs. 25.4, *p*= 0.49) (Supplementary Figure S3A,B).

Current smokers had a longer rwPFS (current 39.8 m vs. never 18.6 m vs. former 13.1 m, *p* = 0.013) and OS (current 50.7 m vs. never 24.9 m vs. former 20.6 m, *p* = 0.31) (Supplementary Figure S4A,B).

**Figure 5:**
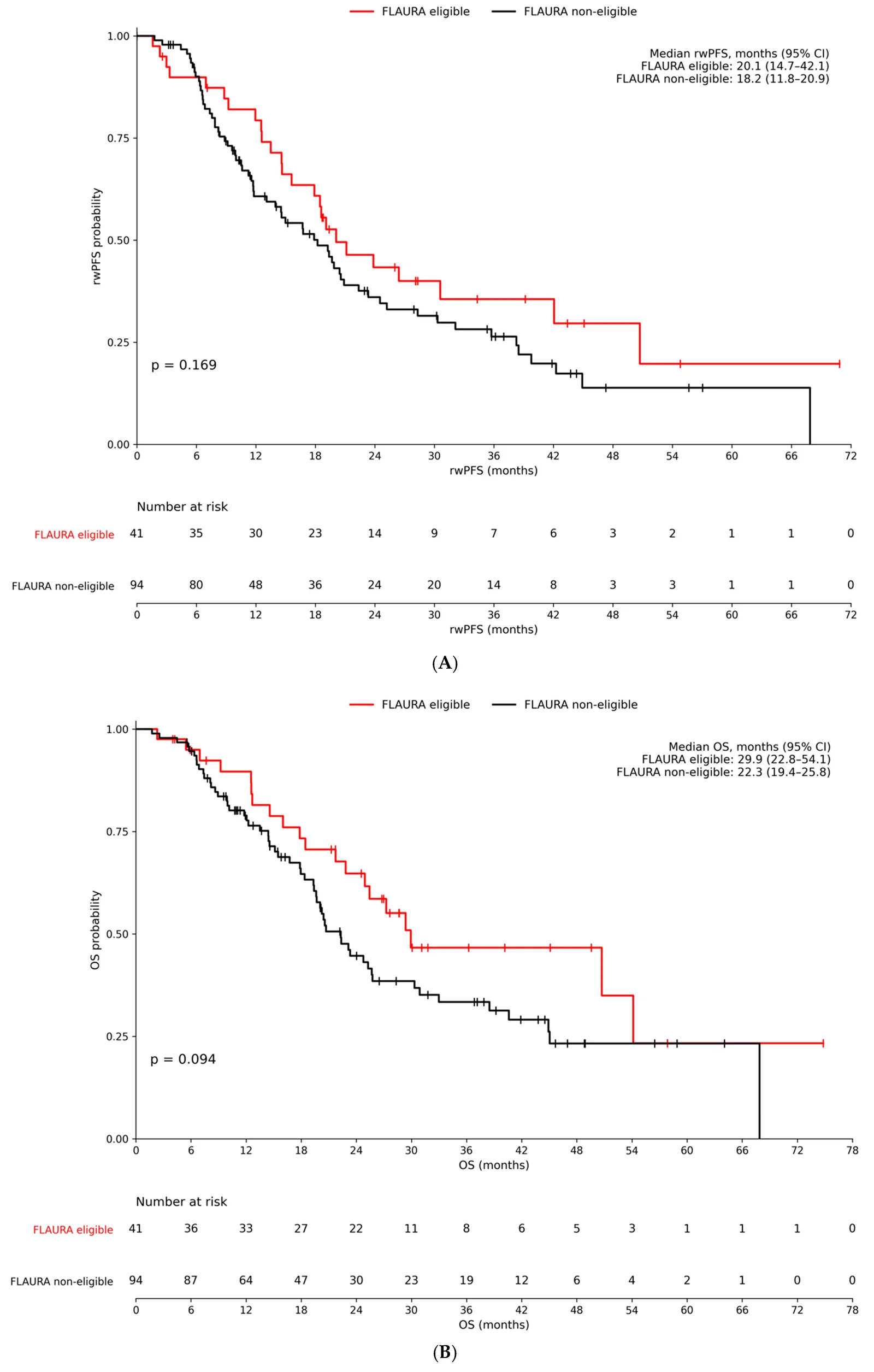
Kaplan–Meier curves representing differences in (**A**) rwPFS and (**B**) OS of patients treated with osimertinib divided according to FLAURA trial eligibility.

When divided per trial eligibility (Figure 5A,B), rwPFS was 20.1 months (14.7–42.1) in the eligible subgroup and 18.2 (11.8–20.9) in the ineligible subgroup. A similar trend was observed for OS, with longer survival in eligible pts (29.9 months, 22.8–54.1) compared with ineligible pts (22.3 months, 19.4–25.8).

## 4. Discussion

We reported a monocentric real-world dataset of patients with EGFR-mutated advanced NSCLC treated with first-line osimertinib and compared their baseline characteristics with those of pivotal RCT cohorts.

Our analysis shows that approximately 70% of patients would not have met the eligibility criteria of each pivotal trial. Similarly to our experience, several other retrospective analyses assessed RW eligibility for the FLAURA trial, reporting ineligibility rates that range from 40% to 55% [8,9,10,12]. The higher proportion of patients considered ineligible in our retrospective experience likely reflects the comprehensive evaluation of all trial inclusion/exclusion criteria, whereas previous studies assessed only a limited set. For example, Wells et al. classified 40% of patients as ineligible for FLAURA but assessed only five criteria (1. ECOG PS, 2. Symptomatic brain metastasis, 3. Hemoglobin levels, 4. Platelet count, 5. Creatinine value) [9]. Importantly, a substantial proportion of patients in our cohort was considered ineligible due to criteria intrinsically related to RCT methodology, such as absence of measurable target lesions, lacking histologic confirmation, or early relapse after curative therapy, rather than true clinical unsuitability for active treatment. This distinction between methodological ineligibility and clinically meaningful ineligibility may partially explain the discrepancy between trial populations and patients routinely treated in clinical practice. When restricting the analysis to clinically meaningful criteria, the proportion of ineligible patients would likely be lower. Across all trials, the most frequent clinically relevant exclusion factors were ECOG PS ≥2, cardiovascular comorbidities, and abnormal laboratory parameters.

**Figure 6:**
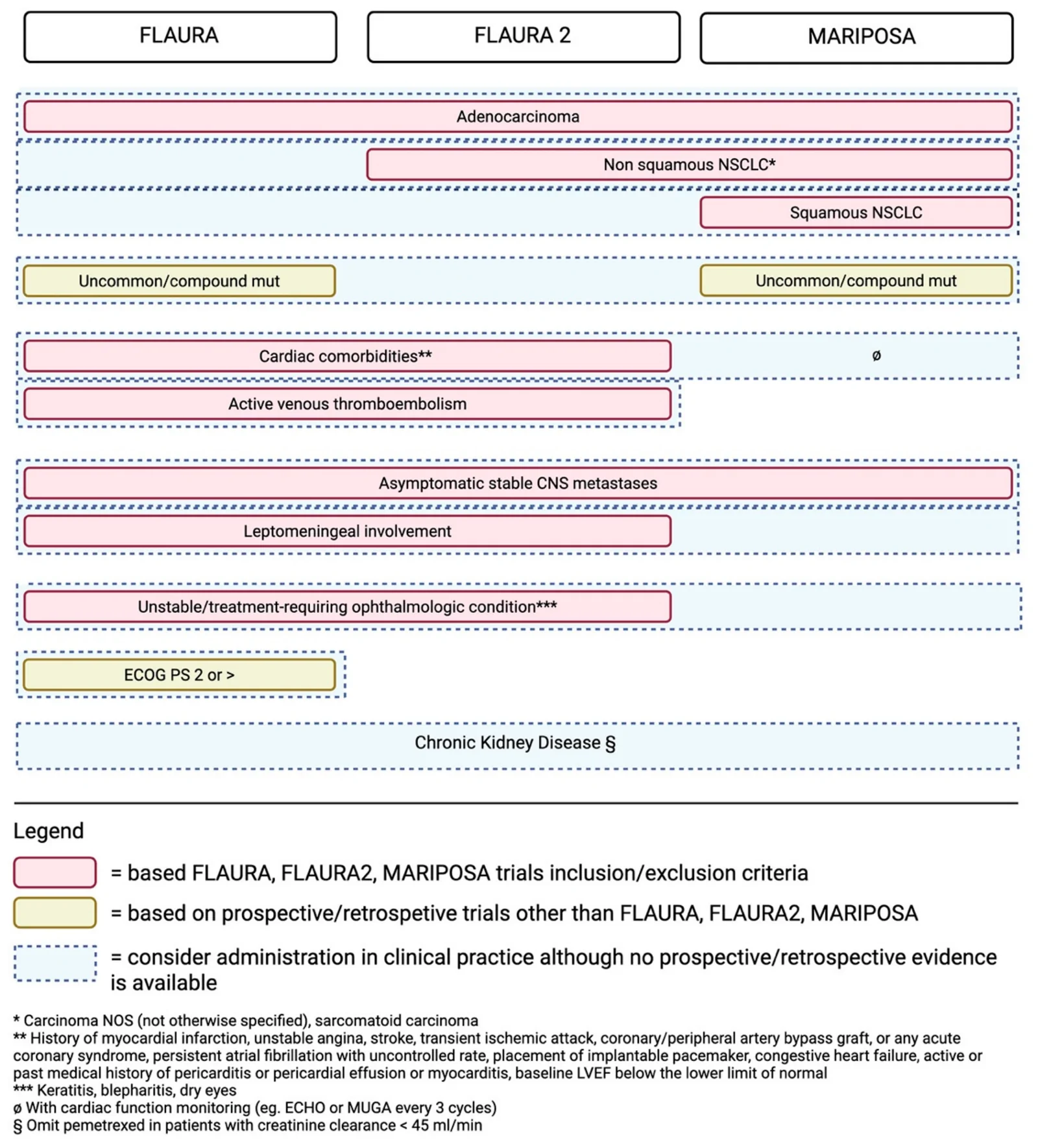
Flow chart for decision-making to support first-line treatment selection in *EGFR*-mutated NSCLCs. Abbreviations: NSCLC = non-small cell lung cancer; mut = mutations; CNS = central nervous system; ECOG PS = Eastern Cooperative Oncology Group Performance Status.

Based on trial eligibility criteria, available evidence, and clinical practice considerations, we propose a pragmatic framework to inform first-line treatment selection in EGFR-mutated NSCLC (Figure 6). This framework is intended to support clinical reasoning rather than serve as a validated treatment-selection algorithm.

Regarding histology, patients with squamous NSCLC were excluded from FLAURA and FLAURA2, but included in MARIPOSA. Although a few case reports have described potential activity of osimertinib in this rare subgroup [13,14], the available evidence remains limited. Therefore, no definitive conclusions can currently be drawn regarding the optimal treatment strategy in this setting.

Patients harboring uncommon *EGFR* mutation were excluded from all three pivotal trials. However, retrospective series [15] as well as early-phase prospective studies [16,17] have demonstrated activity of both osimertinib and amivantamab-based combinations. The UNICORN phase II non-randomized clinical trial reported an ORR of 55.0% and a median PFS of 9.4 months (95% CI, 3.7–15.2) in patients with uncommon single or compound *EGFR* mutations treated with first line osimertinib [16]. Similarly, the ORR of patients treated with first line amivantamab plus lazertinib in the Cohort C of the CHRYSALIS-2 study was 57% with a median PFS of 19.5 months (95% CI, 11.2 to NE) [17]. A phase II trial assessing combination of osimertinib in combination with platinum-based chemotherapy is ongoing in Asiatic population [18]. These findings indicate that targeted therapies have clinical activity in patients with uncommon *EGFR* mutations, although the shorter PFS reported in this setting compared with that observed in trials enrolling patients with common *EGFR* mutations raises uncertainty regarding the durability of disease control. Current evidence does not support preferential use of one regimen over another in this setting, and treatment selection should rely on individual clinical characteristics and treatment feasibility. Moreover, although trial eligibility is frequently based on histological diagnosis and tissue-based *EGFR* testing, determination of *EGFR* status on liquid biopsy has shown to reduce diagnostic delays and improve access to personalized therapies in RW setting [19,20]. Accordingly, treatment decisions are frequently based on ctDNA or cytological samples when tissue specimens are unavailable or insufficient.

Cardiovascular exclusion criteria were substantially more stringent in MARIPOSA than in FLAURA or FLAURA2. Consequently, patients with significant cardiac comorbidities may be more suitable candidates for osimertinib-based strategies, while amivantamab plus lazertinib could still be considered with careful cardiac monitoring. Similarly, given the known thrombotic risk associated with amivantamab [21] and the exclusion of patients with basal active thrombosis in MARIPOSA, osimertinib-based regimens should be favored in patients with ongoing venous thromboembolism.

Patients with spinal cord compression, leptomeningeal disease, or symptomatic brain metastases are well represented in our RW population but excluded from RCTs. In patients with asymptomatic CNS disease, both FLAURA2 and MARIPOSA demonstrated improved outcomes with combination strategies. PFS of patients with baseline CNS involvement in FLAURA2 was 24.9 months (osimertinib + chemotherapy) vs. 13.8 months (osimertinib); similarly, in MARIPOSA, amivantamab plus lazertinib prolonged PFS versus osimertinib (18.3 vs. 13.0 months; HR 0.69) [5]. Therefore, combination approaches may be considered in patients with CNS involvement, particularly in fit patients with high intracranial disease burden. Regarding patients with leptomeningeal disease, these were excluded from the MARIPOSA trial, while longer median duration of exposure with osimertinib plus chemotherapy versus monotherapy was reported in patients with leptomeningeal involvement enrolled in FLAURA2 (25.2 vs. 12.0 months) [22]. In this setting, osimertinib with or without platinum-based chemotherapy may represent a reasonable option according to patients’ general conditions and treatment tolerability [23,24,25]. Notably, amivantamab plus lazertinib was evaluated in a phase II trial in previously treated patients with leptomeningeal disease showing an ORR of 33% and a PFS of 7.8 months, indicating a potential antitumor activity in this subset of patients [26].

Patients with ECOG PS ≥ 2 were excluded from all three trials; however, several retrospective experiences including ours suggest that these patients may derive meaningful benefit from active treatment with osimertinib monotherapy [27]. Combination treatment may not be feasible in this subgroup, although selected patients whose PS impairment is mainly driven by tumor burden could potentially benefit from more intensive approaches.

Laboratory-based exclusion criteria differed among trials, yet the clinical impact of these differences is uncertain. In routine clinical practice, treatment feasibility is generally guided by the severity of laboratory impairment and the overall clinical context rather than by rigid categorical cut-offs alone.

Lastly, patients with conditions such as other active concurrent malignancies, uncontrolled diabetes, or HIV infection, would have been excluded from all the pivotal trials. These findings further highlight the gap between RCT populations and real-world patients, and support the need for individualized therapeutic decision-making.

As previously reported in other studies, we confirmed that patients with risk factors, including ECOG PS ≥ 2, liver or brain metastasis, and age over 65 years old, have worse PFS and OS than those without [10,27,28]. In our cohort, female patients had a longer survival, confirming the role of gender as a significant factor influencing cancer-specific survival [29]. Conversely, TP53 co-mutations did not significantly affect survival outcomes in our series [30], although this finding may reflect the limited number of patients with available TP53 status. A previous study demonstrated that PD-L1 ≥ 50% is associated with an increased risk of progression or death in *EGFR*-mutated NSCLC treated with first-line osimertinib [31]; a similar trend toward worse outcomes was observed in our cohort, although without statistical significance.

These findings suggest that patients with established adverse prognostic factors may represent a subgroup in which treatment intensification could be explored. In this context, preliminary results from the phase III TOP trial demonstrated improved PFS with platinum-based chemotherapy plus osimertinib versus osimertinib alone in patients harboring TP53 co-mutations. Similarly, exploratory analyses from MARIPOSA suggested that amivantamab plus lazertinib may mitigate the negative prognostic impact of high-risk features, including TP53 co-mutations, detectable baseline ctDNA, lack of ctDNA clearance, and liver metastases [32].

An individual patient data comparison of FLAURA2 and MARIPOSA demonstrated a significant PFS advantage favoring the osimertinib-plus-chemotherapy regimen, although this difference was not confirmed for OS. Furthermore, exploratory analyses suggested a greater benefit with the FLAURA2 regimen in patients with baseline CNS metastases, L858R mutations, or absence of liver metastases [33]. However, these findings should be interpreted cautiously given the intrinsic limitations and potential biases of indirect cross-trial comparisons.

In addition, a minority of patients in our cohort had previously received curative-intent treatment for early-stage disease before developing advanced disease. Previous studies in EGFR-mutated NSCLC treated with osimertinib have reported differences in survival outcomes between patients with recurrent metastatic disease and those presenting with de novo advanced disease [34,35].

rwPFS in our cohort was consistent with outcomes reported in the FLAURA experimental arm and in the control arms of FLAURA2 and MARIPOSA, as previously observed in other RW studies [8], confirming the reproducibility of RCT results in real-world settings when osimertinib monotherapy is administered. However, in our cohort, OS was shorter than the one reported in the RCT, potentially due to trial-ineligible patients in our cohort as well as patients with negative prognostic factors. Other studies have previously demonstrated a significantly longer OS and PFS in the FLAURA-eligible group when compared to ineligibles [10,27]. Similarly, PFS and OS in our study were numerically higher among eligible patients, suggesting that treatment in ineligible patients is still feasible, though less effective.

Both combination regimens are associated with higher rates of all-grade and grade ≥ 3 adverse events compared with osimertinib monotherapy. However, the toxicity profiles differ substantially between regimens. Hematologic and gastrointestinal toxicities were more common with osimertinib plus chemotherapy [3], whereas cutaneous toxicity, paronychia, infusion-related reactions, and venous thromboembolism were more frequently observed with amivantamab plus lazertinib [5,6]. Therefore, baseline comorbidities, expected toxicity profile, and patient preferences should all be considered when selecting the optimal first-line strategy.

Although both FLAURA2 and MARIPOSA demonstrated improved outcomes over osimertinib alone, cost-effectiveness remains an important consideration for healthcare systems. Available analyses suggest that combination regimens prolong quality-adjusted life years compared with osimertinib monotherapy, but are associated with substantially higher financial costs and toxicity-related burden [36,37]. Direct comparative pharmacoeconomic analyses between FLAURA2 and MARIPOSA are currently lacking.

Another unresolved issue concerns the optimal management of patients who relapse after adjuvant osimertinib or after consolidation osimertinib following chemoradiotherapy [38,39,40]. Given the absence of prospective evidence in this setting, treatment decisions should currently be individualized. Potential options may include osimertinib rechallenge, platinum-based chemotherapy, continuation of osimertinib beyond progression, or amivantamab-based combinations according to prior treatment exposure and timing of relapse.

Lastly, the selection of first-line treatment should also take into account the potential therapeutic sequence and the availability of subsequent-line options, with the aim of optimizing long-term disease control throughout the patient journey. However, given the rapidly evolving treatment landscape and the lack of mature comparative sequencing data, no definitive recommendations regarding the optimal treatment sequence can currently be made.

This study has several limitations. Firstly, although the trial inclusion/exclusion criteria have been extensively assessed, some criteria may present bias due to the intrinsic nature of a retrospective study. This may have led to an underestimation of the number of ineligible patients, although this is unlikely to impact significantly the findings of our study. Furthermore, we did not analyze toxicity experienced by the patients during the treatment, which could have revealed a different safety profile in eligible and non-eligible patients [34,35]. Moreover, as per clinical practice, we did not assess ctDNA at different timepoints; such data could have enriched translational research and confirmed the prognostic role of basal ctDNA in *EGFR*-mutated NSCLC. Lastly, the single-center design may limit the generalizability of our findings to other real-world settings. Multicenter studies involving more diverse patient populations are warranted to assess the external validity of our results.

## 5. Conclusions

In conclusion, FLAURA2 and MARIPOSA multidrug regimens currently represent the best first-line strategy for *EGFR*-mutated advanced or metastatic NSCLCs, although osimertinib monotherapy can be considered a valid therapeutic option in a selected subgroup of patients. However, the absence of a direct comparison between the three regimens, in terms of survival outcome, toxicities, QoL, and cost-effectiveness, make it impossible to define a strict decision-making flow chart. In this panorama, eligibility-based profiling may represent a pragmatic and clinically useful framework to support personalized first-line treatment decisions in *EGFR*-mutated NSCLC.

## Figures and Tables

**Table 1:** Patients’ characteristics (*n* = 135).

Patients’ Characteristics	*N* = 135
Age at diagnosis–years	
Median [IQR]	67 [57–76]
Range	38–92
Male sex–no. (%)	45 (33)
Smoking status–no. (%)	
Never	65 (48)
Current	26 (19)
Former	39 (29)
N/A	5 (4)
Histology–no. (%)	
Adenocarcinoma	127 (94)
Adenosquamous carcinoma	3 (2)
Squamous cell carcinoma	4 (3)
Sarcomatoid NSCLC	1 (<1)
Stage at diagnosis–no. (%)	
IA	1 (<1)
IB	7 (5)
IIA	0 (0)
IIB	5 (4)
IIIA	7 (5)
IIIB	8 (6)
IIIC	3 (2)
IVA	12 (9)
IVB	92 (68)
*EGFR* mutation type–no. (%)	
Exon 19 deletion	74 (55)
L858R	46 (34)
Other	15 (11)
*EGFR* mutation determination–no. (%)	
Histological sample	116 (86)
Cytological sample	11 (8)
ctDNA	8 (6)
PD-L1-no. (%)	
<1%	52 (39)
1–49%	45 (33)
≥50%	20 (15)
N/A	18 (13)
Stage at Osi start–no. (%)	
IVB	101 (75)
IVA	25 (19)
IIIC	2 (1)
IIIB	5 (4)
Local recurrence	2 (1)
Distant metastasis at Osi start–no. (%)	
<3 sites	87 (64)
≥3 sites	48 (36)
Brain	48 (36)
Liver	23 (17)
Bone	77 (57)
Lung	44 (33)
Adrenal gland	12 (9)
Pleural effusion	33 (24)
Other	47 (35)
ECOG PS at Osi start–no. %	
0	46 (34)
1	65 (48)
2–3	23 (17)
N/A	1 (<1)

Abbreviations: IQR = interquartile range; no = number; N/A = non-available; NSCLC = non-small cell lung cancer; Osi = osimertinib; ctDNA = circulating tumor DNA; ECOG PS = Eastern Cooperative Oncology Group Performance Status.

**Table 2:** Baseline characteristics comparison between trials population and RW cohort.

	FLAURA-Osi (N = 279)	FLAURA-1ST GEN TKI (N = 277)	FLAURA2-CT + Osi (N= 279)	FLAURA2-Osi (N = 278)	MARIPOSA-AMI + LAZ (N = 429)	MARIPOSA-Osi (N = 429)	RW–Osi (N = 135)
Age-years							
Median	64	64	61	62	64	63	67
Range	26–85	35–93	26–83	30–85	25–88	28–88	38–92
Male sex–no. (%)	101 (36)	105 (38)	106 (38)	109 (39)	154 (36)	178 (41)	45 (33)
Smoking status–no. (%)							
Never	182 (65)	175 (63)	N/A	N/A	299 (70)	295 (69)	65 (48)
Current	8 (3)	9 (3)	N/A	N/A	130 (30) YES	134 (31) YES	26 (19)
Former	89 (32)	93 (34)	N/A	N/A			39 (29)
N/A	0	0	N/A	N/A			5 (4)
ECOG PS–no. %							
0	112 (40)	116 (42)	104 (37)	102 (37)	141 (33)	149 (35)	46 (34)
1	167 (60)	160 (58)	174 (62)	176 (63)	288 (67)	280 (65)	65 (48)
2–3	0	0	1 (<1)	0	0	0	23 (17)
N/A	0	1 (<1%)	0	0	0	0	1 (<1)
Overall disease classification-no. (%)							
Metastatic/Recurrent	264 (95)	262 (95)	265 (95)	271 (97)	N/A	N/A	128 (95)
Locally advanced	14 (5)	15 (5)	14 (5)	7 (3)	N/A	N/A	7 (5)
N/A	1 (<1)	0	0	0	N/A	N/A	0
History of brain metastases—no. (%)	53 (19)	63 (23)	116 (42)	110 (40)	178 (41)	172 (40)	48 (36)
*EGFR* mutation type-no. (%)							
Exon 19 deletion	175 (63)	174 (63)	169 (61)	168 (60)	258 (60)	257 (60)	74 (55)
L858R	104 (37)	103 (37)	106 (38)	107 (38)	172 (40)	172 (40)	46 (34)
Both Exon 19 deletion and L858R	0	0	3 (1)	1 (<1)	0	0	0
N/A	0	0	1 (<1)	2 (1)	0	0	0
Other	0	0	0	0	0	0	15 (11)

Abbreviations: no = number; N/A = non-available; ECOG PS = Eastern Cooperative Oncology Group Performance Status; Osi = osimertinib; 1ST–2ND GEN TKI = first–second generation tyrosine kinase inhibitor (gefitinib and erlotinib); CT = platinum-based chemotherapy; AMI + LAZ = amivantamab + lazertinib; RW = real world.

## Data Availability

The raw data supporting the conclusions of this article will be made available by the authors on request.

## Supplementary Materials

The following supporting information can be downloaded at: https://www.mdpi.com/article/10.3390/cancers18213480/s1, Table S1: Description of disease stage and curative treatment performed for patients with limited-stage disease at diagnosis; Table S2: Frequency of unmet inclusion and exclusion criteria in (A) FLAURA trial, (B) FLAURA2 trial and (C) MARIPOSA trial; Figure S1: Kaplan Meier curves representing (A) rwPFS and (B) OS of patients treated with osimertinib with ECOG PS 0-1 compared to ECOG PS ≥ 2; (C) rwPFS and (D) OS of patients treated with Osimertinib with liver metastasis compared to those without liver localizations; (E) rwPFS and (F) OS of female vs male patients; (G) rwPFS and (H) OS of patients treated with osimertinib with EGFR common mutation compared to uncommon EGFR mutation; Figure S2: Kaplan Meier curves comparing (A) rwPFS and (B) OS of patients treated with osimertinib with age ≥ 65 and age < 65; (C) rwPFS and (D) OS of patients treated with osimertinib with and without brain metastasis; (E) rwPFS and (F) OS of patients treated with osimertinib with and without concurrent TP53 mutations; Figure S3: Kaplan Meier curves comparing (A) rwPFS and (B) OS of patients treated with osimertinib with PD-L1 ≥ 50% and PD-L1 0-49%; Figure S4: Kaplan Meier curves comparing (A) rwPFS and (B) OS of patients treated with osimertinib according to smoking status.
