# Supplementary material for: Real-World Eligibility for FLAURA, FLAURA2, and MARIPOSA in Patients with EGFR-Mutated Advanced NSCLC: A Retrospective Single-Center Study
Source: Cancers (Basel). 2026 Oct 28;18(21):3480. doi: 10.3390/cancers18213480 (PMC13761898; doi:10.3390/cancers18213480)
Supplement: Supplementary file 1 [file cancers-18-03480-s001.zip › cancers-4621437-supplementary.pdf]

Supplementary Table S1. Description of disease stage and curative treatment performed for patients with limited-stage disease at diagnosis

Stage at diagnosis – no. (%)	N=23
IA	1 (4)
IB	7 (30)
IIA	0
IIB	5 (23)
IIIA	7 (30)
IIIB	3 (13)
Curative treatment type – no. (%)	
Sequential CTRT	4 (18)
RT	2 (9)
Surgery	9 (39)
Neoadjuvant chemotherapy + surgery	1 (4)
Neoadjuvant chemotherapy + surgery + RT	1 (4)
Surgery + adjuvant chemo(immuno)therapy	4 (18)
Perioperative chemoimmunotherapy + Surgery + RT	1 (4)
Neoadjuvant chemotherapy	1 (4)
Median time from curative treatment end to metastatic disease – months [IQR]	1,4 [0.5-3.3]

Abbreviations: no = number; CTRT = chemoradiotherapy; RT = radiotherapy; IQR = inter quartile rate;

Supplementary Table S2: Frequency of unmet inclusion and exclusion criteria in (A) FLAURA trial, (B) FLAURA2 trial and (C) MARIPOSA trial

A

**FLAURA TRIAL**		Number of patients excluded
**INCLUSION CRITERIA**	**1: Provision of informed consent**	NE
	**2: Age ≥ 18 years old**	0
	**3: Adenocarcinoma histology**	5
	**4: Advanced or metastatic stage**	0
	**5:** *EGFR* Ex19del, L858R, either alone or in combination with other *EGFR* mutations	15
	**6: Provision of tumor tissue sample**	19
	**7: Treatment naïve for advanced NSCLC**	0
	**8: ECOG PS0-1**	23
	**9: Presence of at least one target lesion**	18
	**10: Female adequate contraceptive measures**	NE
	**11: Male adequate contraceptive measures**	NE
	**12: Provision of informed consent for genetic research**	NE
**EXCLUSION CRITERIA**	**1: Involvement in the planning or conduction of the study**	NE
	**2:**	7
	Treatment naïve for advanced NSCLC	0
	Major surgery within 4 weeks of first dose of Osi	7
	Radiotherapy treatment more than 30% of bone marrow within 4 weeks of first dose of Osi	0
	Patients unable to stop concurrent use of P450 (CYP) 3A4 inhibitors/inducers	0
	**3: Concurrent malignancy requiring systemic treatment within 2 years of first dose of Osi**	4
	**4: Unresolved toxicity from prior systemic therapy**	0
	**5: Spinal cord compression or symptomatic and unstable brain metastases requiring steroids**	31
	**6: Uncontrolled systemic disease including hypertension, active bleeding diathesis, active HBV/HCV/HIV**	3
	**7: Refractory nausea, vomiting, chronic gastrointestinal disease, significant bowel resection**	1
	**8: Cardiac criteria**	17
	QTc interval > 470	0

	Any clinically important abnormalities in rhythm, conduction, or morphology of resting ECG, e.g., complete left bundle branch block, third-degree heart block, second-degree heart block, PR interval >250 msec.	2
	Any factor that increased the risk of QTc prolongation (concomitant medications known to prolong QT interval, congenital QTc syndrome)	16
	**9: History of ILD or radiation pneumonitis which required steroid treatment**	1
	**10: Unpaired blood exams**	8
	Neutrophil count <1.5 x 10^9/L	0
	Platelets count <100 x 10^9/L	1
	Hemoglobin <90 g/L	1
	Alanine transaminase increased >2.5x the ULN if no demonstrable liver metastases or >5xULN in the presence of liver metastases	5
	Aspartate transaminase increased >2.5x the ULN if no demonstrable liver metastases or >5xULN in the presence of liver metastases	2
	Total bilirubin >1.5xULN if no liver metastases or >3xULN in the presence of documented Gilbert's Syndrome (unconjugated hyperbilirubinemia) or liver metastases	0
	Creatinine >1.5xULN concurrent with creatinine clearance <50 mL/min	1
	**11: Breast feeding**	0
	**12: History of hypersensitivity to Osi**	0
	**13: Judged by the Investigator that the patient should not participate in the study**	NE
	**14: Prior allogenic bone marrow transplantation**	0

Abbreviations: NE = Not Evaluable; NSCLC = non-small cell lung cancer; ECOG PS = Eastern Cooperative Oncology Group Performance Status; Osi = osimertinib; HBV = hepatitis B virus; HCV = hepatitis C virus; HIV = human immunodeficiency viruses; ILD = interstitial lung disease; ULN = limit of normal

B

**FLAURA2 TRIAL**		Number of patients excluded
**INCLUSION CRITERIA**	**1: Capable of giving informed consent**	NE
	**2: Provision of informed consent**	NE

	**3: Provision of informed consent for genetic testing**	NE
	**4: Age ≥ 18 years old**	0
	**5: Non-squamous NSCLC histology**	4
	**6: Advanced or metastatic stage (IIIB-IVB)**	0
	**7: *EGFR* Ex19del, L858R, either alone or in combination with other *EGFR* mutations**	15
	**8: Provision of tumor tissue sample**	19
	**9: Treatment naïve for advanced NSCLC and systemic treatment for early-stage disease completed at least 12 months prior to treatment start**	7
	**10: ECOG PS0-1**	23
	**11: Life expectancy > 12 months**	0
	**12: Presence of at least one target lesion**	18
	**13: Female adequate contraceptive measures**	NE
	**14: Male adequate contraceptive measures**	NE
**EXCLUSION CRITERIA**	**1: Spinal cord compression or symptomatic and unstable brain metastases requiring steroids**	31
	**2: History of ILD or radiation pneumonitis which required steroid treatment**	1
	**3: Uncontrolled systemic disease including hypertension, active bleeding diathesis, active HBV/HCV/HIV**	3
	**4: Cardiac criteria**	17
	QTc interval > 470	0
	Any clinically important abnormalities in rhythm, conduction, or morphology of resting ECG, e.g., complete left bundle branch block, third-degree heart block, second-degree heart block, PR interval >250 msec.	2
	Any factor that increased the risk of QTc prolongation (concomitant medications known to prolong QT interval, congenital QTc syndrome)	16
	**5: Unpaired blood exams**	19
	Neutrophil count < LLN	1
	Platelets count < LLN	4
	Hemoglobin <90 g/L	1
	Alanine transaminase increased >2.5xULN if no demonstrable liver metastases or >5xULN in the presence of liver metastases	5

	Aspartate transaminase increased >2.5xULN if no demonstrable liver metastases or >5xULN in the presence of liver metastases	2
	Total bilirubin >1.5xULN if no liver metastases or >3xULN in the presence of documented Gilbert's Syndrome (unconjugated hyperbilirubinemia) or liver metastases	0
	Creatinine clearance <60 mL/min	10
	**6: Concurrent malignancy requiring systemic treatment within 2 years of first dose of treatment**	4
	**7: Unresolved toxicity from prior systemic therapy**	0
	**8: Refractory nausea, vomiting, chronic gastrointestinal disease, significant bowel resection**	1
	**9: Treatment naïve for advanced NSCLC and systemic treatment for early-stage disease completed at least 12 months prior to Osi start**	7
	**10: Prior treatment with anti *EGFR* TKIs**	0
	**11: Major surgery within 4 weeks of first dose of treatment**	7
	**12: Radiotherapy treatment more than 30% of bone marrow within 4 weeks of first dose of treatment**	0
	**13: Patients unable to stop concurrent use of P450 (CYP) 3A4 inhibitors/inducers**	0
	**14: Participation in another clinical trial during the 4 weeks prior to treatment start**	0
	**15: Involvement in the planning or conduction of the study**	NE
	**16: Judged by the Investigator that the patient should not participate in the study**	NE
	**17: Prior treatment allocation or randomization in this study**	NE
	**18: Currently pregnant or breast-feeding**	0
	**19: History of hypersensitivity to Osi or drugs with similar structure**	0
	**20: Contraindication for pemetrexed and cisplatin/carboplatin**	0
	**21: Prior allogenic bone marrow transplantation**	0

Abbreviations: NE = Not Evaluable; NSCLC = non-small cell lung cancer; ECOG PS = Eastern Cooperative Oncology Group Performance Status; ILD = interstitial lung disease; HBV = hepatitis B virus; HCV = hepatitis C virus; HIV = human immunodeficiency viruses; LLN = lower limit of normal; ULN = upper limit of normal; TKIs = tyrosine kinase inhibitors; Osi = osimertinib.

C

MARIPOSA TRIAL		Number of patients excluded
INCLUSION CRITERIA	**1: Age ≥ 18 years old**	0
	**2: Histological or cytologically confirmed, locally advanced or metastatic NSCLC**	0
	**3: *EGFR* Ex19del, L858R, either alone or in combination with other *EGFR* mutations**	15
	**4: Provision of tumor tissue sample**	19
	**5: Unresolved toxicity from prior systemic therapy**	0
	**6: Presence of at least one target lesion**	18
	**7: Unpaired blood exams**	12
	Hemoglobin ≥ 10 g/dL	5
	Neutrophil count ≥ 1.5 x 10^9/L	0
	Platelets count ≥ 75 x 10^9/L	1
	Alanine transaminase and aspartate transaminase ≤ 3 ULN	2
	Total bilirubin ≤ 1.5xULN (participants with Gilbert's syndrome can enroll if conjugated bilirubin is within normal limits)	2
	Creatinine <1.5 x ULN (or if available, creatinine clearance >45 mL/min)	4
	**8: ECOG PS0-1**	23
	**9: Provision of informed consent**	NE
	**10: Woman of childbearing potential must have a negative serum or urine pregnancy test**	NE
	**11: Female adequate contraceptive measures**	NE
	**12: A woman must agree not to donate eggs for the purposes of assisted reproduction**	NE
	**13: Male adequate contraceptive measures**	NE
	**14: Male participant must agree not to donate sperm for the purpose of reproduction**	NE
	**15: Participant must be willing and able to adhere to the lifestyle restrictions specified in this protocol**	NE
EXCLUSION CRITERIA	**1: Treatment naïve for advanced NSCLC and systemic treatment for early-stage disease completed at least 12 months prior to treatment start**	7
	**2: Symptomatic brain metastases or receiving low-dose corticosteroid treatment (≤10 mg/day prednisone or equivalent) for at least 2 weeks prior to treatment start**	30

	**3. Active or past medical history of leptomeningeal disease**	4
	**4: Spinal cord compression that has not been definitively treated with surgery or radiation or requires steroid treatment within 2 weeks prior to**	5
	**5: Uncontrolled tumor related pain**	3
	**6: History of ILD or radiation pneumonitis which required steroid treatment**	0
	**7: Uncontrolled illness**	11
	Uncontrolled diabetes	2
	Ongoing or active infection	3
	Active bleeding diathesis	0
	Impaired oxygenation requiring continuous oxygen supplementation	0
	Refractory nausea and vomiting, chronic gastrointestinal diseases, inability to swallow the formulated product, or previous significant bowel resection	1
	Psychiatric illness	1
	Any ophthalmologic condition that is either clinically unstable or requires treatment	4
	**8: Active malignancy**	3
	**9: Cardiovascular criteria**	27
	Deep vein thrombosis or pulmonary embolism within 1 month prior to treatment start	11
	Any of the following within 6 months prior to randomization: myocardial infarction, unstable angina, stroke, transient ischemic attack, coronary/peripheral artery bypass graft, or any acute coronary syndrome	2
	QTc interval > 470	0
	Atrial fibrillation with uncontrolled rate	0
	Complete left bundle branch block, third- or second-degree heart block, PR interval >250msec	1
	Placement of implantable cardioverter defibrillator	1
	Congenital long QT syndrome, family history of long QT syndrome, or unexplained sudden death under 40 years of age, or any concomitant medications known to prolong QT interval	16
	Uncontrolled hypertension	0
	Congestive heart failure	0
	Active or past medical history of pericarditis, pericardial effusion, or myocarditis	0

	Baseline LVEF below the lower limit of normal	0
	**10: History of hypersensitivity to of amivantamab, lazertinib, or osimertinib,**	0
	**11: Patients unable to stop concurrent use of P450 (CYP) 3A4 inhibitors/inducers**	0
	**12: Prior treatment with anti *EGFR* TKIs**	0
	**13: Patient has received an investigational medication within 12 months before randomization or is currently enrolled in an investigational study**	0
	**14: Pregnant, breast-feeding, or planning to become pregnant women**	0
	**15: Men planning to father a child**	0
	**16: Judged by the Investigator that the patient should not participate in the study**	NE
	**17: Active HBV,HCV or other hepatitis**	4
	**18: Active HIV infection**	0
	**19: Major surgery within 4 weeks of first treatment administration**	7

Abbreviations: NSCLC = non-small cell lung cancer; NE = Not Evaluable; ULN = upper limit of normal; ECOG PS = Eastern Cooperative Oncology Group Performance Status; ILD = interstitial lung disease; LVEF = Left Ventricular Ejection Fraction; TKIs = tyrosine kinase inhibitors; HBV = hepatitis B virus; HCV = hepatitis C virus; HIV = human immunodeficiency viruses.

Supplementary Figure S1: Kaplan Meier curves representing (A) rwPFS and (B) OS of patients treated with osimertinib with ECOG PS 0-1 compared to ECOG PS ≥ 2; (C) rwPFS and (D) OS of patients treated with Osimertinib with liver metastasis compared to those without liver localizations; (E) rwPFS and (F) OS of female vs male patients; (G) rwPFS and (H) OS of patients treated with osimertinib with *EGFR* common mutation compared to uncommon *EGFR* mutation;

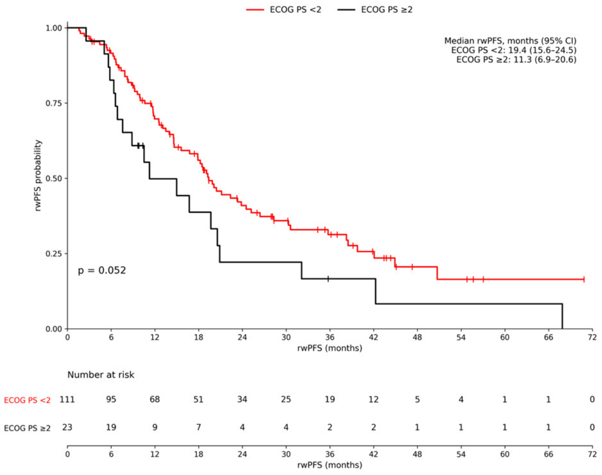

(**A**)

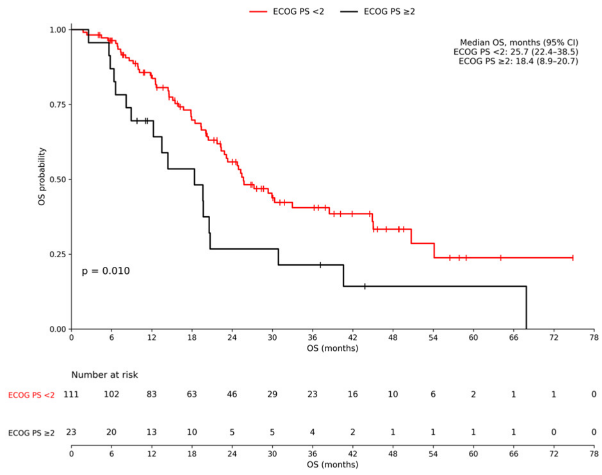

(**B**)

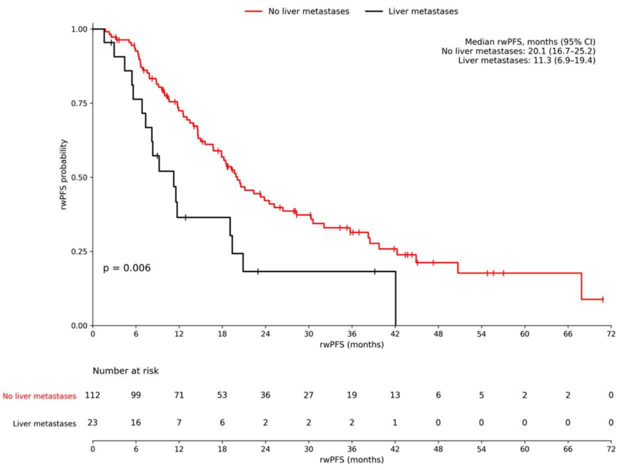

(**C**)

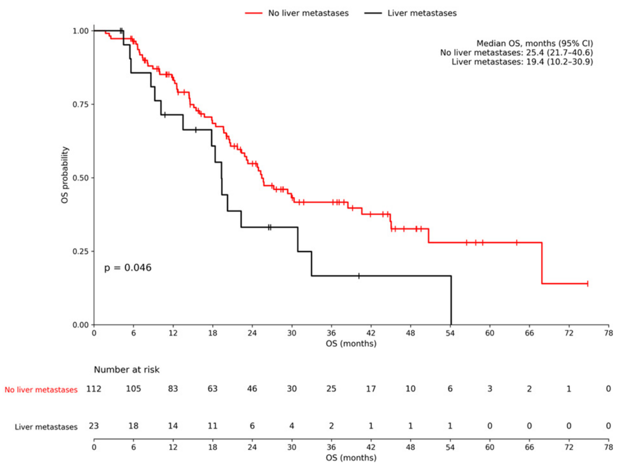

(**D**)

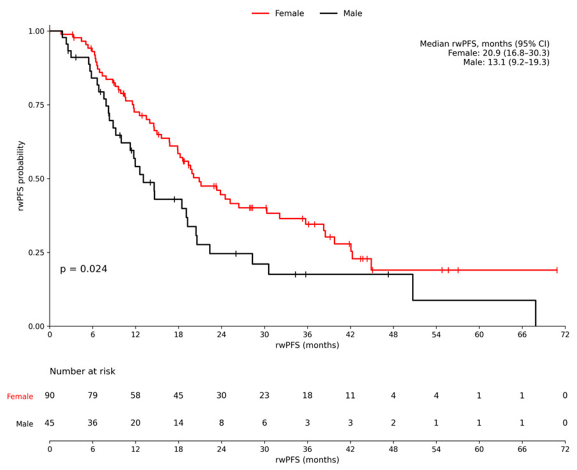

(**E**)

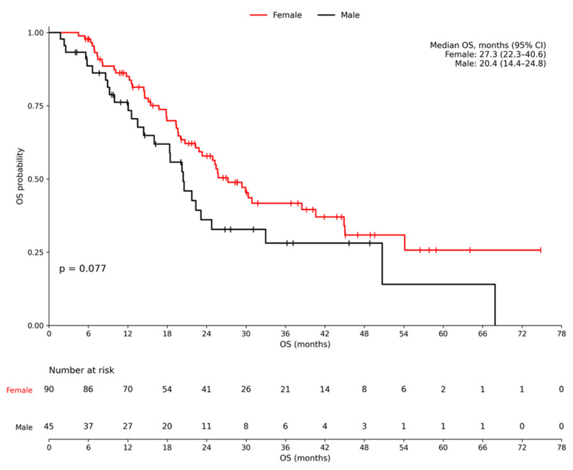

(**F**)

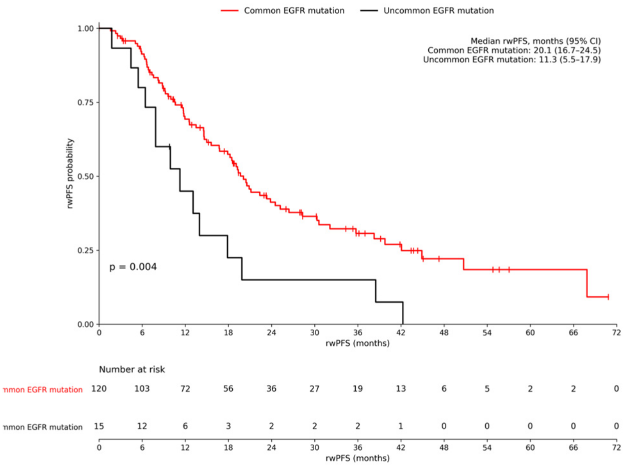

Common EGFR mutation
Uncommon EGFR mutation
Median rwPFS, months (95% CI)
Common EGFR mutation: 20.1 (16.7–24.5)
Uncommon EGFR mutation: 11.3 (5.5–17.9)
rwPFS probability
p = 0.004
rwPFS (months)
Number at risk
mmon EGFR mutation 120 103 72 56 36 27 19 13 6 5 2 2 0
mmon EGFR mutation 15 12 6 3 2 2 2 1 0 0 0 0 0
0 6 12 18 24 30 36 42 48 54 60 66 72
rwPFS (months)

(**G**)

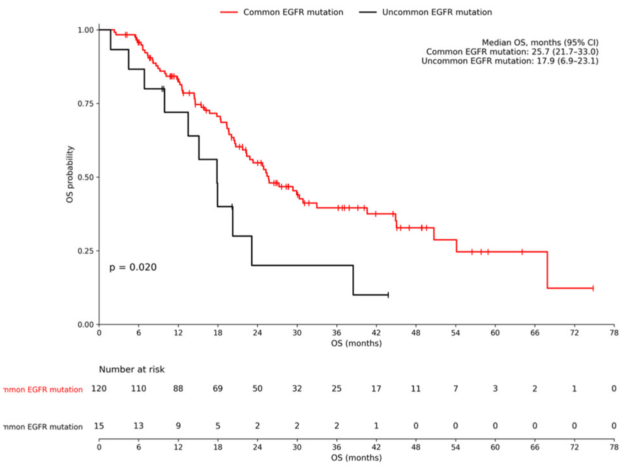

Common EGFR mutation
Uncommon EGFR mutation
Median OS, months (95% CI)
Common EGFR mutation: 25.7 (21.7–33.0)
Uncommon EGFR mutation: 17.9 (6.9–23.1)
OS probability
p = 0.020
OS (months)
Number at risk
mmon EGFR mutation 120 110 88 69 50 32 25 17 11 7 3 2 1 0
mmon EGFR mutation 15 13 9 5 2 2 2 1 0 0 0 0 0 0
0 6 12 18 24 30 36 42 48 54 60 66 72 78
OS (months)

(**H**)

Supplementary Figure S2: Kaplan Meier curves comparing (A) rwPFS and (B) OS of patients treated with osimertinib with age ≥ 65 and age < 65; (C) rwPFS and (D) OS of patients treated with osimertinib with and without brain metastasis; (E) rwPFS and (F) OS of patients treated with osimertinib with and without concurrent TP53 mutations.

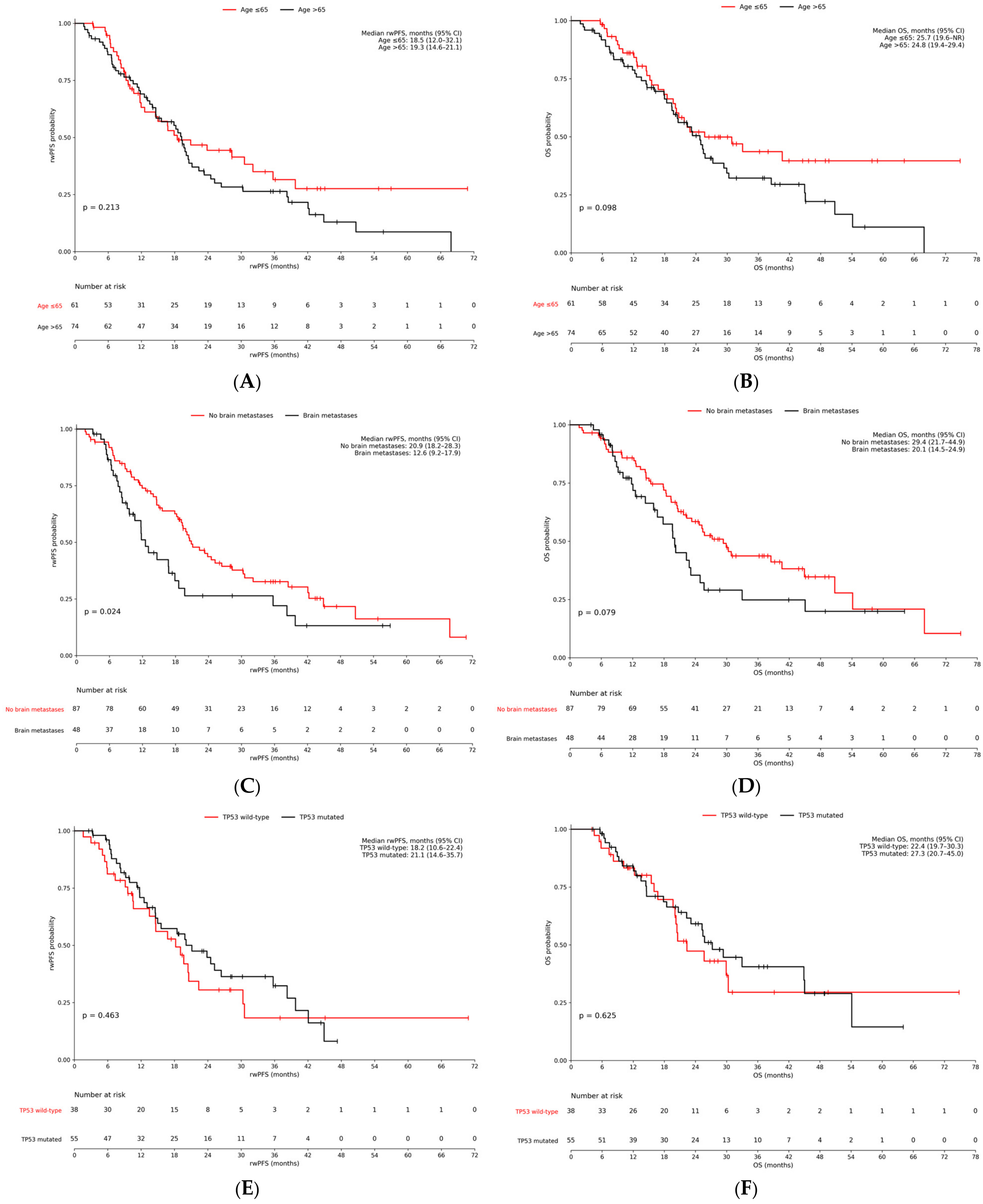

Supplementary Figure S3: Kaplan Meier curves comparing (A) rwPFS and (B) OS of patients treated with osimertinib with PD-L1 ≥ 50% and PD-L1 0-49%

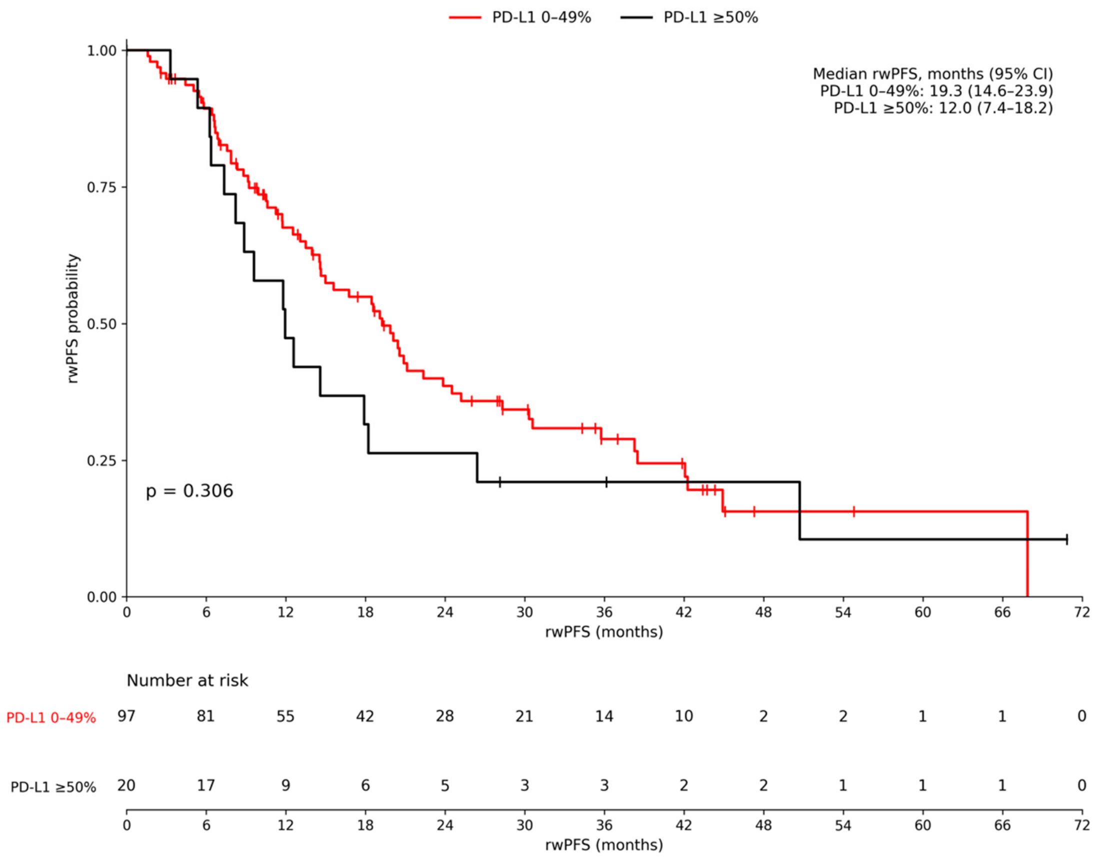

(**A**)

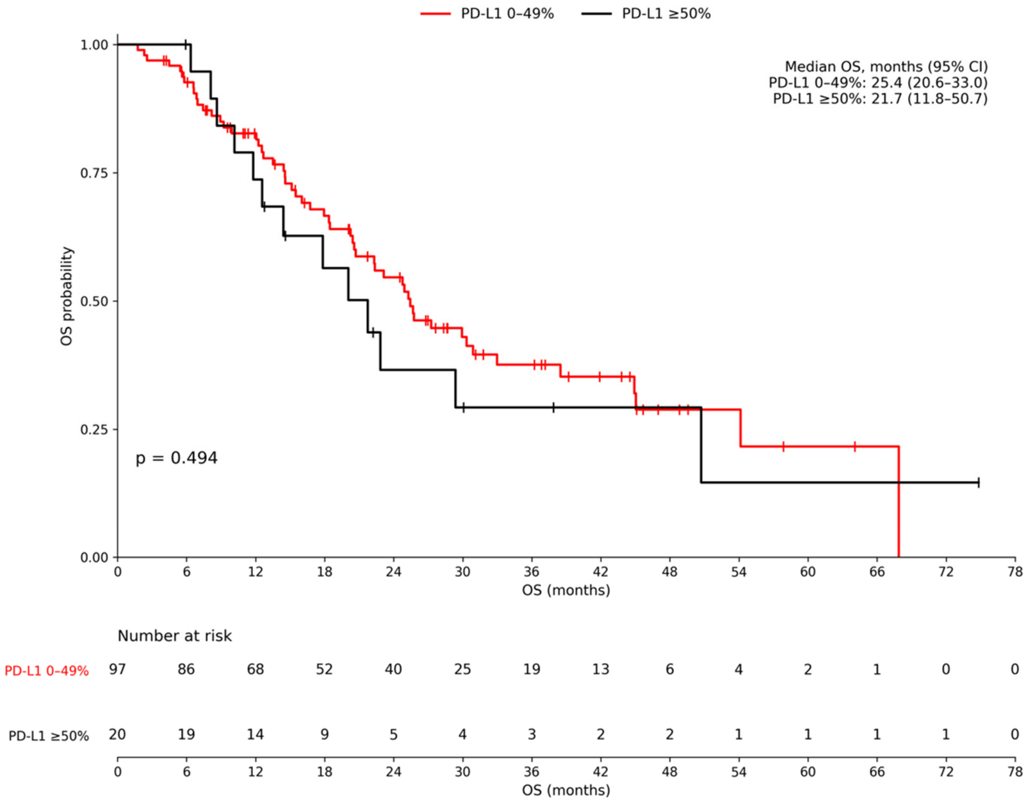

(**B**)

Supplementary Figure S4: Kaplan Meier curves comparing (A) rwPFS and (B) OS of patients treated with osimertinib according to smoking status

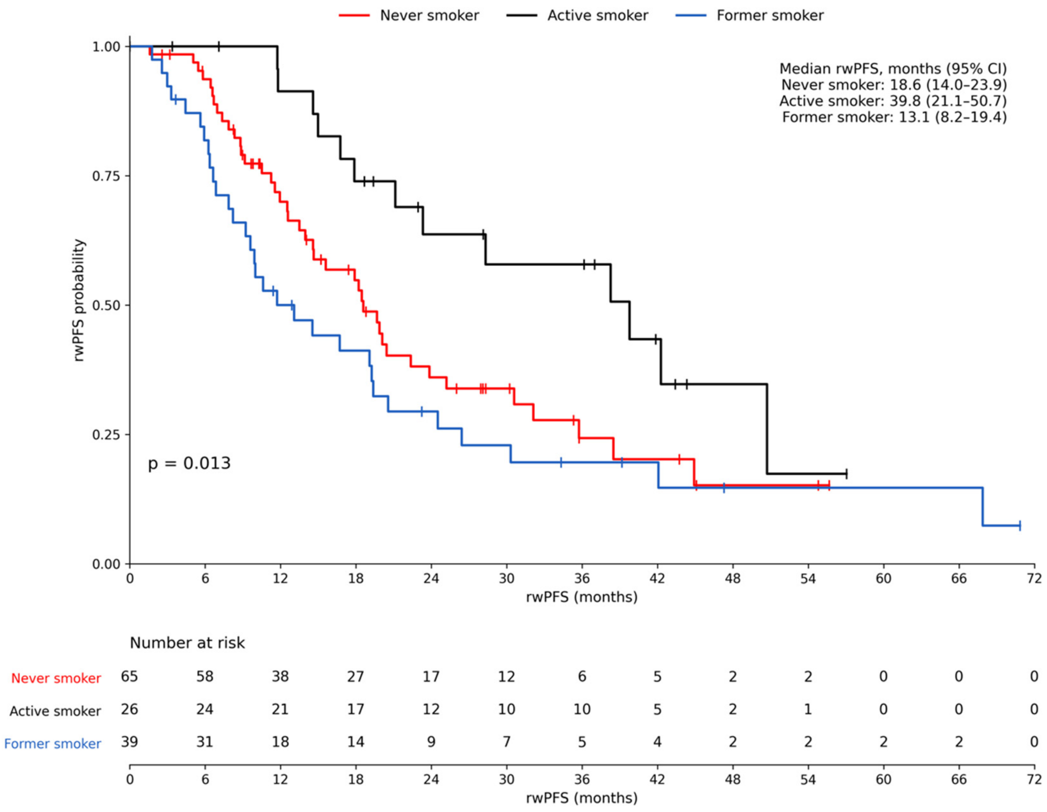

(A)

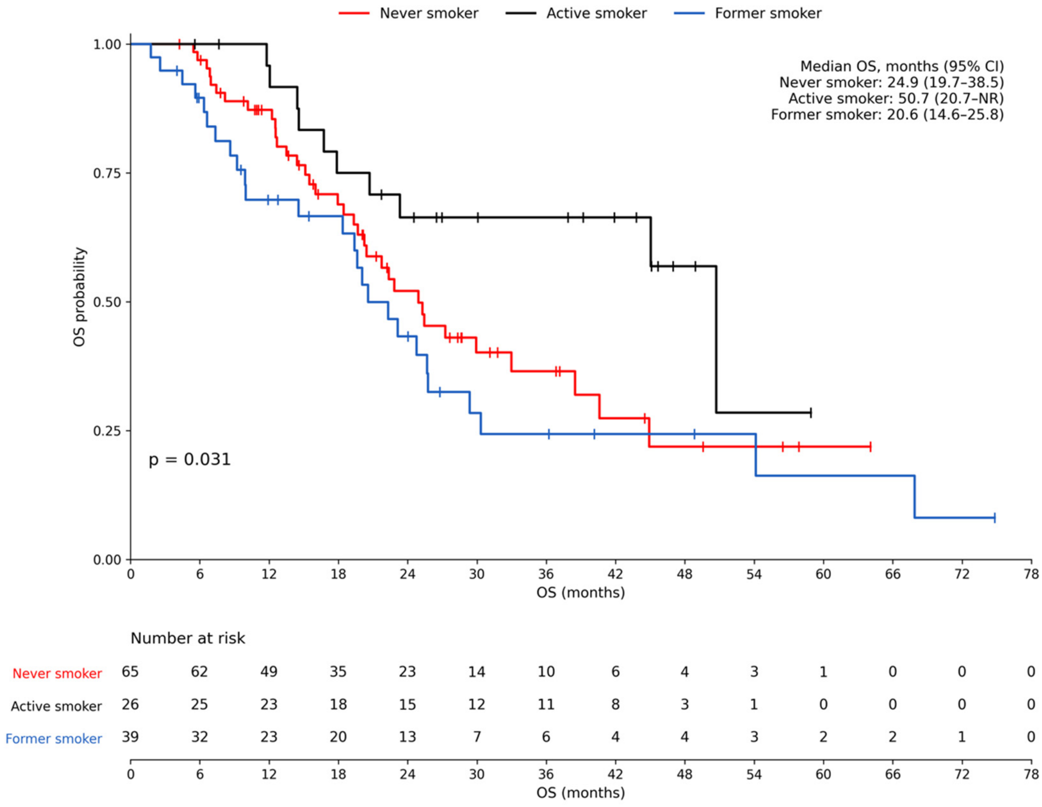

(B)
